# Reasons rural Laotians choose home deliveries over delivery at health facilities: a qualitative study

**DOI:** 10.1186/1471-2393-12-86

**Published:** 2012-08-28

**Authors:** Vanphanom Sychareun, Visanou Hansana, Vatsana Somphet, Sisouvanh Xayavong, Alongkone Phengsavanh, Rebecca Popenoe

**Affiliations:** 1 Faculty of Postgraduate Studies, University of Health Sciences, Vientiane, Lao PDR; 2 Department of Public Health, Global Health Division and Department of Neurobiology, Care Sciences and Society, Division of Nursing, Karolinska Institutet, SE-171 77, Stockholm, Sweden; 3 Dean of the Faculty of Postgraduate Studies, University of Health Sciences, Vientiane City, Lao PDR

## Abstract

**Background:**

Maternal mortality among poor rural women in the Lao People’s Democratic Republic (Lao PDR) is among the highest in Southeast Asia, in part because only 15% give birth at health facilities. This study explored why women and their families prefer home deliveries to deliveries at health facilities.

**Methods:**

A qualitative study was conducted from December 2008 to February 2009 in two provinces of Lao PDR. Data was collected through eight focus group discussions (FGD) as well as through in-depth interviews with 12 mothers who delivered at home during the last year, eight husbands and eight grandmothers, involving a total of 71 respondents. Content analysis was used to analyze the FGD and interview transcripts.

**Results:**

Obstacles to giving birth at health facilities included: (1) Distance to the health facilities and difficulties and costs of getting there; (2) Attitudes, quality of care, and care practices at the health facilities, including a horizontal birth position, episiotomies, lack of privacy, and the presence of male staff; (3) The wish to have family members nearby and the need for women to be close to their other children and the housework; and (4) The wish to follow traditional birth practices such as giving birth in a squatting position and lying on a “hot bed” after delivery. The decision about where to give birth was commonly made by the woman’s husband, mother, mother-in-law or other relatives in consultation with the woman herself.

**Conclusion:**

This study suggests that the preference in rural Laos for giving birth at home is due to convenience, cost, comfort and tradition. In order to assure safer births and reduce rural Lao PDR’s high maternal mortality rate, health centers could consider accommodating the wishes and traditional practices of many rural Laotians: allowing family in the birthing rooms; allowing traditional practices; and improving attitudes among staff. Traditional birth attendants, women, and their families could be taught and encouraged to recognize the signs of at-risk pregnancies so as to be able to reach health facilities on time.

## Background

The Lao People’s Democratic Republic (Lao PDR) is a low-income country with 5.6 million people belonging to 49 ethnic groups. Almost half of the female population is made up of women aged 15 to 49, but the reproductive health status of women and girls, especially in remote areas, remains poor. The 2005 Lao Reproductive Health Survey found a total fertility rate (TFR) of 4.07 children per woman aged 15–49 years old. In rural areas, however the TFR is higher due to an earlier age of marriage and shorter intervals between births [1].

Lao PDR also has the highest levels of maternal mortality in the Western pacific Region at 405 per 100,000 live births in 2005 [2]. The target of Millennium Development Goal 5 (MDG), established in 1990, is to reduce the global maternal mortality ratio (MMR) by three-quarters by 2015 [3], meaning a reduction to185 per 100,000 births in Lao PDR by 2015. A further reduction to a MMR of 166 per 100,000 live births by 2020 has been set within Lao PDR as a National Target [4]. Current health indicators suggest that Lao PDR has a long way to go to meet both MMR goals.

One of the main factors contributing to high MMR and perinatal mortality is lack of access to and low use of maternity services [5]. The main causes of maternal death in Lao PDR are obstructed labor, sepsis, and postpartum hemorrhage [6], all of which are due in large part to poorly equipped and poorly financed health services; insufficient knowledge about reproductive health among women; and lack of modern contraceptive methods. In addition, delays in decision-making by pregnant women, delays in getting to a health center, and delays in treatment have been linked to the high MMR [7].

With the assistance of the World Health Organization, Lao PDR initiated the Safe Motherhood Program in 1998 to reduce maternal mortality and morbidity [7]. This program seeks to guarantee the rights of women to receive health services, at all stages of life, and to make sure newborns also receive health services. The main components of the program are antenatal care services (ANC), safe delivery services, post-partum check-ups, management of complications at all stages, and family planning services [7]. In addition, with technical assistance from WHO and UNFPA the Ministry of Health (MOH) launched a Maternal, Newborn, and Child Health Initiative (MNCH) in 2008 in some provinces. The aims of the MNCH initiative are: 1) Governance and management capacity building at district level; 2) Improved information systems at the local level; 3) Interprofessional training of healthcare workers to improve quality; 4) Provision of integrated outreach services; 5) Improvement of physical infrastructure; 6) Community mobilization; and 7) Empowerment of village health volunteers [8].

The 2005 Lao Reproductive Health Survey found that among children born in the last 5 years, approximately 85% of births occurred at home [1]. Of the 15% that took place at a health facility, 1.8% were at central hospitals, 5.1% at provincial hospitals, 4.8% at districts hospitals, less than 1% at health centers and 0.3% at private clinics. Most strikingly, while 51.2% of women living in urban areas gave birth at a health facility, only 9.8% of rural women with road access and 2.1% of women living in rural areas without road access did. Reasons rural women gave for not giving birth at a health facility were that there was no need (75%), that they were too far away (34%), and that the costs were too high (5-6%) [1]. As many studies elsewhere have also shown, the place of delivery in Lao PDR is usually decided on by family members, and not by the pregnant woman alone [9-12].

This study aimed to add depth to the 2005 Lao Reproductive Health Survey [1] results by exploring why rural women in particular so seldom give birth at health facilities. There has been little research on how cultural and social traditions among non-Lao ethnic groups influence childbirth practices, or on what other factors might influence the decision-making process about where to give birth in rural Lao PDR. The ultimate goal is that this information be incorporated into health interventions and education programs in order to reduce the high MMR and Infant Mortality Rate (IMR) within the country.

## Methods

### Study site

The study was conducted from December 2008 to January 2009 in two rural communities, one in the central Laotian province of Khammouane and one in the southern province of Champasack. In each province the local health office helped select two districts, one “intermediate” and one “remote” in terms of their ease of access to health facilities. This purposive sampling also took into account whether information on home deliveries from the previous year was available. All the selected villages had road access, but road conditions were often poor.

Khammouane province has a population of 333,487 made up predominantly of lowland and upland Lao groups, but also including non-Lao Phuan, Tahoy, Kri, and Katang ethnic groups, as well as some others. In 2007 the estimated total fertility rate (TFR) was 5.0; the number of children born per woman was 4.6; the IMR was 103, and the MMR was 420 per 100,000 live births [13]. Champasack province has a population of 603,880 and an estimated TFR of 4.2; the average number of children born per woman, however, was 4.8. The IMR was 70.5 and the MMR was 320 per 100,000 live births [13].

### Participants

In order to locate participants we turned to district hospitals and health centers to help us locate villages where many women had had home deliveries. Once we had chosen the villages, the health centers as well as village health volunteers (VHVs) and the Lao women’s organization (LWU) assisted us in finding and recruiting women by choosing randomly from lists of women who had had home deliveries compiled by local officials.

We first carried out eight FGDs with a total of 43 participants and then chose some FGD participants as well as some additional village women for in-depth interviews. In our selection process we sought to include women from ethnic Lao as well non-Lao ethnic groups and to include women who had had a range of pregnancy outcomes, such as obstructed labor or stillbirth, or who had relatives who had had such experiences. Once the women were chosen their husbands and mothers were also recruited to the study in order to gain a fuller understanding of the decision-making processes behind home deliveries.

The key informants who were interviewed included 12 women who had had a home delivery during the past year, eight husbands and eight mothers of the women who had recently delivered.

### Data collection

FGDs and IDIs were used to gain a better understanding of how rural women and their families reasoned about giving birth at home versus at a health facility. FGDs were used to explore the social context of home births and to capture community norms surrounding decision-making related to childbirth [14]. FGDs also allowed the exploration of differing views among participants through the discussions they entailed. The interviews were used to learn about personal experiences related to home delivery, including decision-making processes.

Four trained interviewers and three field assistants were recruited and trained to collect information in the study areas. The same semi-structured interview guide was used in both the FGDs and in the interviews (Table 1), and both were carried out in the Lao language. Before the FGDs, the moderator (either the first or second author) introduced all participants, explained the general topics to be discussed and let participants know that everyone was invited to contribute their ideas. The moderator was assisted by two research assistants, one taking notes and another making observations. The FGDs were recorded using a voice recorder. After the FGDs, the note-taker and the moderator reviewed their hand-written notes together while listening to the recording. FGDs were held either in the village temple or at the office of the village head.

**Table 1:** Guideline for FGDs and in-depth-interviews

1.	Socio demographic characteristics (age, residence, education, occupation, income, marital status, religion, parity)
2.	Please tell us about your last pregnancy and any problems during the last pregnancy (gravida, parity, ANC, problems)?
3.	Where did you go to give birth to your last child? Why? Why or why not did you delivered at the health facilities?
4.	Who assisted you during the delivery? What did they do during the labor? Was there use of traditional medicines? Which ones?
5.	How (and why) did you decide give birth at home? Who decided where you would give birth? Why?

The interviews were held in a quiet place in the participants’ homes. Each informant was interviewed once and the interviews lasted between 1 and 2 hours. Interview questions dealt with cultural practices around childbirth; decision-making about place of delivery; gender aspects of childbirth practices; and the reasons for home delivery. One IDI and one FGD were performed each day so that emerging issues could be incorporated into subsequent FGDs and interviews.

### Data analysis

Recordings from the IDIs and FGD’s were used to generate transcripts in the Lao language. The transcripts were then cross-checked with the recordings by the research team and analyzed using content analysis [15]. The investigators read the transcripts many times in order to become familiar with the subjects raised, and then coded the transcripts according to emergent categories. The principal investigator initiated the coding process, and the other authors then added their own views of the codings and together they reached a consensus. After completion of the coding process, categories and major themes were developed and classified. Information gleaned from the IDIs and FGDs was compared to form an overall picture of participant perceptions and attitudes, including areas where there was a diversity of views [16].

### Ethical clearance

This research proposal was approved by the Ethical Committee for Health Research of the University of Health Sciences, in Lao PDR. All participants in interviews and FGDs were informed about the nature of the study and gave verbal consent before participation in the study. They were assured anonymity and privacy, and were informed that they could withdraw at any time.

## Results

### Participants

Participants in the FGDs ranged in age from 18 to 37 years. The socio-demographic characteristics of women in the FGDs are shown in Table 2. Most of them had some primary education but about one third of them were illiterate. A little less than half of the women in the FGDs (41.5 %) held traditional animist beliefs and the others were Buddhists. Almost all were farmers. The parity ranged from one to seven and the women’s most recent intrapartum experience had occurred between 1 and 12 months previously.

**Table 2:** Characteristics of FGD participants

**No of participants**	**Age range (years)**	**Parity range**	**Most recent intrapartum experience**
5	18-35	1-5	4 weeks to 8 months ago
6	19-37	1-6	14 weeks to 12 months ago
6	18-34	1-6	18 weeks to 11 months ago
5	18-36	1-7	12 weeks to 8 months ago
7	19-34	1-4	15 weeks to 12 months ago
6	19-34	1-5	4 weeks to 10 months ago
7	20-31	1-4	14 weeks to 12 months ago
8	18-33	1-5	15 weeks to 12 months ago

The women who participated in in-depth interviews (IDI) ranged in age from 17 to 38 years, had husbands between 26 and 46 years old, and mothers and mothers-in-law between 45 and 50 years old. About 37.5 % of husbands and 50 % of mothers were illiterate. The characteristics of the key informants who participated in IDIs are summarized in Table 3.

**Table 3:** Socio-demographic characteristics of participants in in-depth interviews

	**Women (12)**	**Husbands (n = 8)**	**Mothers/**
			**Mothers in-law (n = 8)**
**Age**	**Range 18–37**	**Range 26–46**	**Range 45–70**
	**Mean = 25.3**	**Mean = 32.87**	**Mean = 52.37**
Religion			
Buddhist	7	5	5
Animist	4	2	3
Christian	1	1	
Marital status			
Married	12	4	6
Widowed	0	0	2
Educational level			
Illiterate	3	3	3
Primary	6	3	5
Lower Secondary	3	1	0
Occupation			
Home duties	1	0	2
Farmer	9	6	6
Self-employed	1	0	0
Other	1	2	0
Obstetric Gynecologic Factors			
Stillbirth	1	Na	Na
Abortion/miscarriage	3	Na	Na

Two women, one in a FGD and one in an interview, had experienced a stillbirth. Two of the women interviewed had had complications: one had had excessive bleeding and the other had lost consciousness during the birth. The woman with bleeding was referred to the provincial hospital and had recovered there. The woman who lost consciousness was assisted by a TBA and recovered without needing to go to hospital. The babies were alive and, aside from the two stillborn children, there were no cases of disabled children or death.

### Overall summary of results

The reasons women and men gave for choosing home deliveries had at least as much to do with the perceived advantages of home births – they are convenient, time-tested, and near to family -- as with the perceived disadvantages of hospital births – they involve high costs, make it difficult for family to be present, and require that women give birth in the disliked horizontal position. Women and men recognized the value of the hospital as a place to turn to when a delivery went wrong, but found the home setting fully adequate and preferable for giving birth otherwise (See Table 4).

**Table 4:** Results: Why do rural Laotians prefer home delivery, and who decides?

**1. Perceived advantages with giving birth at home**	
·	Ease, convenience and nearness to family
·	Desirable birthing practices at home: TBA’s, birthing position, and traditional postpartum practices
·	Previous positive experiences
**1. Perceived disadvantages with giving birth at health facilities**	
·	Costs and transport
·	Dislike of position of delivery and medical procedures
·	Presence of male birth attendants
·	Lack of privacy and confidentiality
·	Previous negative experiences
**1. Reasons for Choosing Delivery at the Health Facilities**	
·	Skilled birth attendants
·	High risk pregnancy
**1. Decision-making about place of delivery**	
·	Husbands and other family members primary decision-makers
·	Influence of mothers and mothers-in-law

### Perceived advantages of home births

*Ease, Convenience, and Nearness to Family:* Ease, convenience, and nearness to relatives were the most cited reasons for home delivery. Family members noted that with home deliveries they did not need to move from one place to another and they and their relatives did not need to go to hospital for visiting.

"*“I preferred to deliver at home because at home it is easier. You can boil the water and cook, and I can accommodate my relatives and husband.” (Woman, 35)*"

"*“I would like my wife to deliver at home because it is easy and cheap; additionally, I can help my wife during labor by massaging her abdomen during pain.” (Husband, 34)*"

The availability of family support was also mentioned by women, mothers and husbands. At home births, mothers and husbands could stay close to the women and provide psychological support as well as physical care, including back massage and gentle touching of the abdomen, soothing the woman in labor and making her feel warm. In hospitals, the husband and other family members are not permitted to enter the delivery room. One husband described how he cared for his wife during labor:

"*“I gave psychological support to my wife when she delivered by putting my hand to her hand and gave her emotional support with pushing during labor” (Husband, 31)*"

Women themselves also saw the presence of their husbands and other family members as an advantage to giving birth at home:

"*“I like to deliver at home because I like my husband staying with me during labor and I feel warmest and I am not afraid. My husband holds my hands and the TBA also stays with me” (Woman, 29)*"

In addition to close relatives, other family members and neighbors also pay visits to women giving birth, and giving birth at a health facility makes this impossible, as one woman pointed out:

"*“It is impossible to drag my family to visit me at the hospital as I have 3 children and my relatives as well....” (Woman, 30)*"

*Desirable Birthing Practices at Home:* Pregnant women wanted their husbands and mothers to be close to them during labor to give psychological support, but were often also attended by a TBA, in addition to other relatives. Women and their families expressed great confidence in TBA’s, their skills and their knowledge. TBAs supervised deliveries either at their own homes or at the woman’s home. The predominant role of TBAs was to give traditional medicine and “magic water” that has been prayed over by a healer or by lay people to the woman in labor to relieve pain, treat abnormal discharge, and give her energy and strength to push the baby out. TBAs helped women during labor by advising them to walk around during early contractions, and by compressing the abdomen during later stages. One 26-year old woman described the TBA’s role thus:

"*“During labor, the TBA suggested that I walk in order to make the head of the baby engaged in my pelvis to make it easy to deliver.”*"

Mothers and husbands also provided care and support to women in labor. Mothers gave herbal medicine and coconut milk to their daughters and put eggs on their daughters’ abdomens, all in order to ease delivery. Husbands helped in similar ways:

"*“I used an herbal medicine called “wane” mixed in water and I put this water on the head of my wife to help make the labor easy and to reduce pain during labor.” (Husband, 32)*"

Participants also mentioned that women in labor normally drank some water that had been blessed with sacred words or had the water put on their abdomen in order to make the birth easier. One TBA noted that,

"*“… With a difficult delivery, I blow water from the spiritual healer or use another method such as soap in the vagina to make it an easy delivery and if it is not successful then I suggest that the woman go the hospital.”*"

TBAs also advised women on different positions during labor in order to facilitate childbirth, and used other traditional remedies to ease delivery:

"*“To support and assist a woman during labor I suggest that she lie down and hold onto a rope and I push her abdomen in order to make the head of the baby come down; then I do a vaginal examination [not as invasive as those done at health facilities] to check the cervical dilatation, and when it’s fully opened I tell her to use her all her energy to push.” (TBA, 38)*"

Position of delivery was an important factor in women’s preference for home delivery. At home women give birth in a sitting position with their knees to their chest, holding onto a rope hanging from the ceiling (described above), a position they prefer to the prone position required at the hospital. In addition, at home women can follow the practices of taking a hot bath and of lying over a fire to warm their bodies during the postpartum period, traditional valued practices thought to help heal the body after delivery. As one 28-year old woman explained,

"*“After delivery at home, I can take a hot bath so the wounds will heal quickly and the bleeding will stop, and I can lie on a hot bed.”*"

Even when TBA’s did not attend a birth, relatives attending the birth saw to it that the practices described above were followed.

*Previous positive experiences:* Previous positive experience with home delivery was another common reason given for choosing to give birth at home. Most women had already delivered at least one child at home, and if this delivery had gone well there was no reason to change. Even if a woman herself had not delivered previously at home, the delivery experiences of mothers-in-law, mothers, aunts and grandmothers all influenced the place of delivery.

### Perceived disadvantages with giving birth at a health facility

*Costs and transport:* In addition to the social and psychological support available to women who give birth at home, giving birth at home is also cheaper, many participants pointed out. Giving birth at home costs little beyond the obligatory payment to the TBA, while giving birth at a health facility involves paying for the delivery itself, any needed medicines, the hospital room, and transport to the health facility, and food. One 29-year old woman expressed the views of many when she said,

"*“I could not afford the hospital delivery due to the high cost of a hospital delivery compared to a home delivery. For the hospital delivery, I had to pay for the rooms and medicine, while for home delivery, I did not pay anything except the gift for TBA’s who assisted my delivery.”*"

Physical distance and lack of transportation were also obstacles to delivering at health facilities. In the rural areas there is no public transport between the villages and the health facilities, so people have to rely on their own means of transport, such as motorbikes and small trucks, to get to health centers. One mother described how transportation difficulties prevented her from seeing to it that her daughter gave birth at the health facility as she, the mother, had hoped:

"*“My daughter was delivering for the first time, so I wanted her to deliver at the health facilities; however, I waited for a bus or pick-up for two hours; there was not any transport and I did not have my own transport.”*"

Sometimes, labor started at night and the family did not want to travel at night; in other cases, families were afraid that labor could start while travelling a long distance to health facilities. In some cases there simply was not time to get to a hospital because of the quick progression of labor.

"*“Because the labor was easy and quick and was a short labor, I couldn’t get to the health facilities on time.” (Woman, 26)*"

*Dislike of position of delivery and medical procedures:* Unlike the squatting position that women preferred, health facilities demand that women lie on their backs on a labor bed with their legs strapped to metal stirrups. Women did not like giving birth in this position:

"*“I don’t like the birthing position in the health facilities where you have to lie with your legs wide open.” (Woman, 28)*"

Some women also noted that they were afraid of some medical procedures commonly performed at the hospital such as episiotomies and suturing after delivery. A 24-year old woman described her dislike of common hospital procedures thus:

"*“I did not want to deliver at a hospital because I was afraid that the health staff would cut me and I would not be able to lie over a fire or on a hot bed and I was afraid of bleeding … The other medical procedure I did not want was a vaginal examination, which they do at the health facilities. When I delivered at home where there was no vaginal examination.”*"

As this woman explains and as mentioned above, another drawback of giving birth at a hospital is that women cannot follow their traditional birthing practices, considered essential for a woman to recover properly from childbirth. As one 28-year old woman reasoned,

"*“If I were to deliver at a hospital, I could not lie on a hot bed immediately after birth because the stitches might come out.”*"

*Presence of male birth attendants:* Another aspect of hospital services that women did not like was that the birth attendant could be a man. Their husbands, interestingly, could accept this, but most of the women felt shy and embarrassed by having a male attendant.

*Lack of privacy and confidentiality:* Informants also reported that they disliked the lack of privacy and confidentiality at the hospital. The presence of many health staff during delivery coupled with women’s shyness at being naked during delivery made hospital deliveries less appealing than home deliveries:

"*“In the health facilities, there are a lot of health staff during the delivery and I am shy, so I prefer to deliver at home” (Woman, 25)*"

*Previous negative experiences:* Previous negative experiences at the health facilities were another reason women and their families chose not to deliver there. Either the women themselves or their mothers described receiving poor service at health facilities when they were ill, and this discouraged them from wanting to give birth there. One mother described how she got worse rather than better when recently treated for an illness at health center:

"*“My daughter did not want to deliver at the hospital because I had a bad experience with the health staff. During my previous illness, I went to hospital and the health staff gave me some injections; however, I got worse, so I did not want my daughter go to hospital.”*"

### Reasons for choosing delivery at the health facilities: skilled birth attendants and high-risk pregnancies

The only reasons informants gave for giving birth at the health facilities were the skill and medical knowledge of trained health professionals and their ability to assist women if complications arose during delivery. If labor was prolonged, there was excess bleeding, or the baby was in a breech presentation, women and their families preferred to give birth under the supervision of trained health workers. As one mother explained,

"*“I wanted my daughter to deliver at hospital because I was concerned for my daughter’s health and I wanted health staff supervising and examining her; I felt confident that they could help the mother and baby in time.” (Mother, 54)*"

Even though men preferred for their wives to give birth at home, they too recognized the advantages of a hospital delivery if there seemed to be any risk factors in the pregnancy:

"*“I talked to my wife and said that if the labor is prolonged or difficult, I will bring her to the hospital.” (Husband, 27)*"

### Decision-making about place of delivery

The place of delivery was decided by the woman’s husband, mother, grandmother or a TBA, together with the woman herself, but it often seemed that the woman herself had the least say in the matter. As one woman put it,

"“*My husband decided where to deliver; however, he also mentioned that it is up to me and I decided to deliver at home.” (Woman, 28)*"

"*“I consulted with my husband about where to deliver and we decided to deliver at home because my previous delivery also was at home.” (Woman, 35)*"

Even if a woman and her husband preferred to have the delivery at a health centre, the mother, mother-in-law, aunt or even neighbors might advise the woman to deliver at home, in accordance with their own past experience of childbirth.

"*“My mother always delivered at home, so she advised me to deliver at home and there nothing went wrong.*” *(Woman, 25)*"

TBAs and health care providers were another source of advice and influence. According to some women, their health providers at ANC left it up to them to decide if they wanted to give birth at the health facility or at home. As one husband reported,

"*“The nurse did not advise my wife to deliver at the hospital when my wife attended antenatal care. She said that my wife could deliver at home.” (Husband, 27)*"

The vast majority of women thus ended up delivering at home with the assistance of a TBA. If the TBA could not help them, the women sought permission from their husbands to go the hospital and get the extra care they needed there.

## Discussion

This study elucidates the many economic, social, cultural, and accessibility factors that explain why rural Laotians choose overwhelmingly to deliver at home rather than at health facilities. Our findings are supported by much previous research, and point the way to some simple changes and some more complex changes that could be implemented to make it more likely that rural families would choose health facilities for giving birth, ultimately lowering the MMR and IMR in Lao PDR.

In addition to the arguably obvious advantages of home births in terms of ease, convenience, and nearness to family, it is clear that the weight of habit should not be ignored. Women’s mothers and grandmothers and all women before them had given birth at home; why should they do it any other way? Although complications in childbirth and preventable deaths do occur with home births, the vast majority of births, it should be remembered, go smoothly, leaving women and their families with the clear impression that little is to be gained by making the often arduous journey to a health center where they will be largely alone, unattended by relatives, and have to submit to a number of unfamiliar and unwelcome birthing practices. In line with previous research on rural birthing preferences in the world [17-20], these disliked aspects of hospital births include episiotomies, lying prone to give birth, having male birth attendants, and exposing their genitals to strangers. These practices made women feel embarrassed and uncomfortable, and sometimes conflicted with traditional beliefs such as the necessity of lying on a hot bed after delivery. Women and families in our study also complained about the lack of privacy at the hospital and discomfort with being examined by male staff. This attitude was also found in the study of Maternal Waiting Homes, leading the authors to suggest that only female staff should carry out vaginal examinations and that privacy of women and their families should be ensured [17].

Also as in previous studies in other countries [21,22], we found that when there was a sudden onset of labor or labor that starts at night, getting to a health facility was at best inconvenient and at worst impossible, making home births the de facto choice. Our study also confirmed the finding of a previous study that women need to be at home to take care of their children and do housework, adding to home delivery’s convenience and appeal [23].

Lack of access to health facilities due to long distances between rural villages and health facilities, poor roads and high transport costs have been identified as a problem in many developing countries [17,24-26], including Lao PDR [21,27,28], as our study also found. A study in southern Lao PDR about barriers to the use of Maternal Waiting Homes (MWH) also found that the cost of transport to the facility, the cost of drugs when there, and the loss of income were the main constraints [17].

Another factor that has been found to discourage women from having their deliveries at health facilities is the perception that the quality of the care and staff is poor, a factor also named by participants in our study. Women and their families are often dissatisfied with the staff attitudes, procedures, and availability of supplies, among other things [29]. A previous study found that not only the choices of home delivery but also delays in seeking medical care when necessary are partly the result of previous negative experience with the health care system [30].

Although TBA’s were respected and valued by participants in our study, the majority had delivered without assistance from skilled TBAs; only husbands and mothers helped women during delivery. This corresponds to statistics in the National Reproductive Health Survey that found that, since 2005 in Lao PDR, 63.4% of babies were delivered with the assistance of relatives compared to only 12.1% with traditional birth attendants. Health professionals assisted in 18.5% of births (8.1% were assisted by a doctor, 3.5% by a nurse, 3% by a midwife and 3.9% by a health worker) [1]. In urban areas, health professionals delivered 63.2% of births compared to 15.3 per cent in rural areas with road and 5.3% in rural areas without roads [1].

Our study also elucidates the decision-making process about where to give birth, in which primarily husbands, mothers, mothers-in-law and grandmothers, but also TBAs and even local health care workers, have strong influence. Our findings are in line with previous research in Nepal and Indonesia [9,10], Tanzania [11] and Malawi [12] that have found household gender dynamics to be a crucial context to decision-making about where to give birth [31]. Our findings are also in line with an international review of decision-making around health care for malaria that found that even when rural women have responsibility for the health status of households, they must consult with husbands, mothers-in-laws, parents and other relatives who have the ultimate decision-making power about seeking care [32]. A contrast to this pattern is in Nepal, where as in rural Lao PDR husbands and parents made decisions about care in the case of delivery complications, but the initial decision about place of delivery was made by a nurse [33].

### Limitations of the study

The key informants included only women who had experienced a home delivery. Hence, the study did not capture the views of women who delivered at hospitals, and who may have had other views on and experiences of cultural childbirth practices in Laos. Instead, this study aimed to highlight cultural beliefs and social practices among ethnic groups in south and central Lao PDR. Initially, we planned to do direct observation of childbirth at home; however, because of time constraints and because there were no home deliveries during the time we were in the field, we were not able to directly observe any deliveries. We felt that the women were open with us, and is evidenced by the many criticisms of health facility birthing practices that emerged. We tried to build good rapport with women and we provided small towels to women to thank them for their valuable time.

## Conclusion

This study offers insights into the factors that make home births the favoured choice of rural Lao women and their families set against the alternative of costly, inconvenient, invasive, and culturally insensitive health facility births. In doing so the study provides an evidence base for improving health facility childbirth services to meet the needs and wishes of rural women and their families. These findings can be used by policy makers, planners, and healthcare professionals to improve delivery services at health facilities by adapting these services to local needs and wishes. Ultimately, improving birthing services to meet the needs of rural populations can help to reduce maternal and perinatal mortality in Lao PDR.

Our study points to several relatively simple changes that could be made to birthing procedures at health facilities to make them more appealing to women and their families:

· Allowing the presence of family members when women give birth

· Allowing traditional birthing practices when they do not conflict with biomedical evidence (putting magic water on the abdomen, massage, sitting over a “hot” bed postpartum)

· Allowing women to give birth in a squatting position when not medically contra-indicated

· Improving confidentiality and privacy

· Decreasing the involvement of male staff in deliveries where possible

· Allowing women to keep their genitals covered as much as possible during labor

Other changes that would make women and their families more likely to choose hospital births, however, will require more time, money and/or political will:

· Better roads

· Better rural transportation [29]

· Changing the attitudes of healthcare staff

Finally, since women and their families are willing to use health facilities if they perceive their pregnancy to have complications, women, their husbands, their mothers, and TBAs should be informed about what signs to look for that may indicate problems with delivery.

It is a shame that rural women are forced to choose between comfort and caring, on the one hand, and the prowess of biomedicine on the other; ideally health facilities would make both essential aspects of the childbirth experience.

## Competing interests

The authors declare that they have no competing interests.

## Authors’ contributions

SV developed the research proposal, designed the instrument, and collected data in the field sites, analyzed and wrote the draft manuscript. RP contributed to the analyzing and finalized the manuscript. SX and VS assisted in the data collection and preliminary data analysis. VH and AP assisted in the survey instrument development, data collection, data analysis and also contributed to the final version of the manuscript. All authors read and approved the final manuscript.

## Pre-publication history

The pre-publication history for this paper can be accessed here:

http://www.biomedcentral.com/1471-2393/12/86/prepub
